# A quantitative analysis of global environmental protection values based on the world values survey data from 1994 to 2014

**DOI:** 10.1007/s10661-018-6949-z

**Published:** 2018-09-15

**Authors:** Qiuhua Li, Bin Wang, Haifeng Deng, Chaoqing Yu

**Affiliations:** 1 Ministry of Education Key Laboratory for Earth System Modeling, Department of Earth System Science, Tsinghua University, Beijing, 100084 China; 2 LASG, Institute of Atmospheric Physics, Chinese Academy of Sciences, Beijing, 100029 China; 3 College of Earth and Planetary Sciences, University of Chinese Academy of Sciences, Beijing, 100049 China; 4 School of Law, Tsinghua University, Beijing, 100084 China

**Keywords:** Environmental protection, Classification, Action, Attitude

## Abstract

The four most recent sub-datasets of the World Value Survey (WVS) database (i.e., WVS3, WVS4, WVS5, and WVS6) contain a total of 25 non-numerical variables of environmental protection values and cover the period from 1994 to 2014. This study utilized these datasets to obtain the spatiotemporal distributions of the values and provided a preliminary analysis of the environmental protection values in different cultural districts. The work includes 4 parts. First, the information from the values included in the 25 variables is subjectively classified into action values and attitude values according to the meanings of the variable labels. Then, quantitative clustering is used to verify the results of the first step. These two steps consistently classify the 25 variables into “action” and “attitude” families. At the third step, all variables are processed as horizontal distributions in terms of the country using the arithmetic mean of the serial numbers chosen by the respondents because these numbers reflect the grade of the behavior or attitude toward environmental protection. A clustering procedure is also included in this step to reconfirm the classification results of the previous two steps. Finally, the two families are quantified using their common factors, which are the first leading modes of the empirical orthogonal function for each family. The multiyear averaged cultural district mean “action” and “attitude” indices are analyzed according to the World Culture Map. The results show that districts with different cultures have very different environmental protection values.

## Introduction

The greenhouse gas (GHG) concentrations in the atmosphere have been increasing since the industrial revolution due to the use of fossil fuels. The globally averaged concentration of carbon dioxide in the atmosphere was 280 ppm when industrialization started in 1750 and increased to 400 ppm by 2013 (WMO 2011; IPCC 2013). Increased carbon dioxide concentrations influence human health and life, and a scientific consensus has been reached that climate change in the most recent half-century was most likely caused by human activities. Environmental and climate change analyses based on human activities should not neglect the driving force of human society. To a great extent, the culture of a society determines if carbon emissions strategies are accepted by the society, and values are the most important factors in studying the influence of culture on environmental protection because values dominate the behaviors of people (Douglas 1966, 1970; Douglas et al. 1998; Douglas and Wildavsky 1982; Adger et al. 2013). Increasing both public willingness and environmental protection awareness is the foundation of emission reduction actions.

There are many studies in the literature on environmental concern or climate change from the aspects of social backgrounds. Numerous studies have examined the effects of socioeconomic factors from multiple dimensions, concentrating on variables such as age, sex, and education (Engel, Pötschke 1998; Hidano and Aritomi 2005; Torgler and García-Valiñas 2007). It has also been found that environmental legislation can affect environmental behavior (Kalantari et al. 2007). Some studies have highlighted the importance of attitude and behavior toward the environment (Vogel 1994; Tarrant and Cordell 2003; Cluck et al. 2003; Gärling et al. 2003; McFarlanc and Boxall 2003). Many studies have elaborated on the cultural dimensions or value-based approach of climate change impacts and adaptation (Albizua 2014; Leiserowitz 2006; Persson et al. 2015; O’Brien 2009). Moreover, many theoretical and empirical approaches have demonstrated that religious views could have an important influence on environmentalism and values (Greeley 1993; Kempton et al. 1995; Eckberg and Blocker 1996). The complicated relationship between environmentalism and religion depends on political, social, and traditionally ethical correlations (Greeley 1993; Guth et al. 1995). All of the above studies examined environmental preferences, including the effects of socio-demographic variables and other socioeconomic factors, by analyzing certain variables or time periods. However, there are limited numerical data on environmental protection values with temporal-spatial distributions that are suitable for academic and scientific research.

The World Values Survey (WVS, http://www.worldvaluessurvey.org/wvs.jsp) that began in 1981 is the largest cross-national investigation of human beliefs and values ever executed. The survey has comprehensively evaluated religion, gender roles, work motivations, democracy, good governance, social capital, political participation, environmental protection, and subjective well-being (Inglehart et al. 2004). Some authors have used the database to analyze the linkages between environmental protection and the attitudes, behavior, or public support from people. Inglehart (1990, 1995, 1997) developed a theoretical model to explain the environmental attitudes of people. The results indicated that both objective conditions and subjective values have substantial impacts on the support for environmental protection, and the studies argued that environmental concern emerged due to a shift from materialist values toward postmaterialist values. The index of environmental protection values that was used in the above research was directly defined as the percentages of responses to different questionnaires with the answers “strongly agree,” “agree,” “disagree,” and “strongly disagree.” Mondéjar-Jiménez et al. (2012) analyzed the importance of individual behaviors, attitudes, and values in relation to the environmental actions of various countries using a structural equations model to compare the covariance structure of environmental factors in terms of values, attitudes, proactiveness, and actions. However, only 10 variables were selected from WVS1-5 in the paper, which is not typical since there are 10 environmental protection variables in WVS3 alone. Most recently, Mondéjar-Jiménez et al. (2018) analyzed the individual environmental values and behaviors of the citizens of North American countries and their differentiation from the behaviors of European citizens. These authors used the new data from the 2015 WVS (i.e., WVS7) but included only two North American countries. YOGO (2011) investigated the willingness to pay for environmental goods and presented ordinary least square (OLS) estimates using the five WVS datasets in 1981–2007, but the study analyzed only 13 African countries.

The following five studies intended to explain the relationship between related factors and environmental protection using one or more variables in a certain period. These studies did not contain all environmental protection variables or the longitudinal data of the datasets. Gelissen (2007) explained popular support for environmental protection using multilevel analysis, i.e., on the individual-level and contextual-level, to analyze the data from the 1999–2000 WVS and European Values Survey (EVS) datasets. Using data from the WVS and EVS datasets for 1990, 1995, and 1999–2000, Torgler and García-Valiñas (2007) investigated the preferences of individuals to prevent environmental damage in Spain, presenting a richer set of independent variables such as political interest and social capital, as well as a time variable. Contorno (2012) built multiple regression models to measure international environmental concern and compare the cosmopolitan values on environmental attitudes using the data drawn from the 2005 WVS, including impact variables such as cosmopolitanism, egalitarianism, postmaterialism, income, education, age, sex, and political ideology. Ma (2012) studied the readiness of a Chinese citizen to pay an environmental tax/fee using a logistic model based on the 2007 WVS data. The results showed that environmental consciousness, development concept, and political trust were the three main factors that influenced the support from people for economic and environmental policies by the government. Using the 2007 WVS data, Zhu (2015) investigated the impact of income, media, gender, and age on environmental concern using OLS estimates and a nested logistic model.

In summary, these studies did not provide a comprehensive spatiotemporal analysis on environmental values. Moreover, no studies have focused on the non-numerical, discontinuousness, and incompletion of the global WVS data in terms of both time and space. Few studies have tried to quantify these non-numerical data in reasonable and scientific ways or solve the problems of discontinuousness and incompletion of the data. Therefore, it is necessary and significant to reasonably convert these non-numerical and discontinuous data into numerical and continuous data using various statistical methods. The focus of this study is to process the global environmental protection values data in the WVS datasets and generate numerical, continuous, and relatively complete values datasets that include the “action” and “attitude” data.

The remainder of this paper is structured into four main sections as follows. The next section introduces the data selected for this study. “Classification and quantification of the WVS data” section focuses on data processing and classification. “Subjective classification” section presents the analysis of the results, which is followed by the “Conclusions” section.

## WVS data

The data source is the WVS, which was introduced above. The data exist in the form of datasets: WVS1 to 6. Table 1 shows the basic information of the WVS data used in this paper and indicates that different WVS datasets cover different countries. Furthermore, different countries in the same dataset and the same country in different datasets have different respondent numbers. Because of the time discontinuity from WVS1 to WVS2, as well as the obviously fewer valid countries in these two datasets (see Table 1), this paper uses only the last four datasets that continuously cover a period from 1996 to 2011. The datasets are the WVS3 (in 1994–1998), WVS4 (in 1999–2004), WVS5 (in 2005–2009), and WVS6 (in 2010–2014), which include a total of 25 variables to describe the judgment of environment values (see Table 2).

**Table 1:** The basic information of the original and pre-processed WVS data

WVS dataset	Period	Country number	Respondent number	Number of valid country	Number of valid respondent
1	1981–1984	20	25,000	10	13,586
2	1989–1993	42	61,000	18	24,558
3	1994–1998	54	77,129	44	37,542
4	1999–2004	41	60,045	41	25,879
5	2005–2009	58	83,975	55	57,903
6	2010–2014	61	90,350	61	77,867

**Table 2:** Variable names, variable labels and value labels in the selected WVS datasets

Dataset	Variable	Variable label	Value label
WVS 3	V33	Membership of voluntary organizations: environmental organization	1-active member; 2-inactive member; 3-not a member
	V38	I would agree to an increase in taxes if the extra money was used to prevent environmental damage	1-strongly agree; 2-agree; 3-disagree; 4-strongly disagree
	V39	I would buy things at a 20% higher price if it helped to protect the environment	1-strongly agree; 2-agree; 3-disagree; 4-strongly disagree
	V40	Environmental problems can be solved without any international agreements to handle them	1-strongly agree; 2-agree; 3-disagree; 4-strongly disagree
	V41	Environment vs. economic growth	1-protecting the environment should be given priority; 2-economic growth and creating jobs should be the top priority
	V42	Environmental action: choose products that are better for the environment	1-have done; 2-have not
	V43	Environmental action: recycle something rather than throw it away	1-have done; 2-have not
	V44	Environmental action: reduce water consumption for environmental reasons	1-have done; 2-have not
	V45	Environmental action: attend meetings, signed petitions aimed at protecting the environment	1-have done; 2-have not
	V46	Environmental action: contribute to an environmental organization	1-have done; 2-have not
	V49	Human beings should master nature vs. coexist with nature	1-human beings should master nature; 2-humans should coexist with nature
WVS 4	V33	Would give part of my income for the environment	1-strongly agree; 2-agree; 3-disagree; 4-strongly disagree
	V34	Increase taxes if the extra money is used to prevent environmental pollution	1-strongly agree; 2-agree; 3-disagree; 4-strongly disagree
	V35	Government should reduce environmental pollution	1-strongly agree; 2-agree; 3-disagree; 4-strongly disagree
	V36	Environment and economic growth	1-protecting environment; 2-economic growth and creating jobs
	V37	Human & nature	1-master nature; 2-coexist with nature
WVS 5	V29	Membership in environmental organization	0-not a member; 1-inactive member; 2-active member
	V104	Environmental vs. economic growth	1-protect the environment; 2-economic growth and creating jobs
	V105	Would give part of my income for the environment	1-strongly agree; 2-agree; 3-disagree; 4-strongly disagree
	V106	Increase taxes if the extra money is used to protect the environment	1-strongly agree; 2-agree; 3-disagree; 4-strongly disagree
	V107	Government should reduce environmental pollution	1-strongly agree; 2-agree; 3-disagree; 4-strongly disagree
WVS 6	V30	Active/inactive membership: environmental organization	0-not a member; 1-inactive member; 2-active member
	V81	Protecting environment vs. economic growth	1-protecting the environment should be given priority; 2-economic growth and creating jobs should be the top priority
	V82	Past 2 years: given money to an ecological organization	1-yes; 2-no
	V83	Past 2 years: participated in a demonstration for the environment	1-yes; 2-no

The WVS cultural maps of the world made by Ronald Inglehart and Christian Welzel (see http://www.worldvaluessurvey.org/WVSContents.jsp) are also used in this study, and these maps provide a comprehensive measurement of all major areas of human concern from religion and politics, to economics and social life. On the cultural maps, each country is positioned according to the values of the population and not geographical location. For example, Australia, Canada, the USA and Great Britain are cultural neighbors, reflecting their relatively similar values despite their geographical dispersion. The two-dimension coordinates dominate the map pictures, with the ordinate entitled “Traditional values versus secular-rational values” and the abscissa labeled “Survival values versus self-expression values”. The maps show where societies are located on these two dimensions. Upward movement on these maps reflects the shift from traditional values to secular-rational values, while rightward movement indicates the change from survival values to self-expression values.

## Classification and quantification of the WVS data

As mentioned in the “Introduction” section, the WVS data are non-numerical and were obtained from the worldwide questionnaire surveys, which are quite inconvenient for related quantitative studies. This study focuses on how to quantify these non-numerical data. A reasonable classification of the WVS data is very important for this purpose. Because the values that dominate the behavior of people are key in studying the influences of culture on carbon emissions, we attempt to classify and quantify the non-numerical variables of the judgments of environmental protection values from the WVS data using various statistical methods. Thus, the new data not only keep their original characteristics but also are suitable for related quantitative studies. Currently, there is very limited research on the quantification of environmental protection values; thus, this attempt is of significance in both theory and application.

Using these data, we analyze the national will for emission reduction and the environmental protection awareness by the public. Using both the meanings of the variables and the hierarchical clustering method, we classify the values data into two categories first subjectively and then quantitatively, which are called “action” and “attitude” (for emissions reduction). The data are quantified based on the original coded data in the selected WVSs using empirical orthogonal function (EOF) decomposition, i.e., the first leading EOF mode of each category is used as its common factor, which defines the action index or attitude index for environmental protection. The detailed description of the processes and steps of classification and quantification is given in the following section.

### Subjective classification

The classification of the WVS variables includes two steps, subjective analysis and quantitative clustering analysis. In the first step, the variables are subjectively classified into two categories according to only the meanings of the variable labels and value labels. Some of the variables listed in Table 2 belong to the category that indicates the “attitude” toward emission reduction, while others belong to the category that reflects the “action” of emission reduction. The “attitude” family includes the variables V40, V41 and V49 in WVS3; V35, V36 and V37 in WVS4; V104 and V107 in WVS5; and V81 in the WVS6. For example, the variable label for V40 in WVS3 is “environmental problems can be solved without any international agreements to handle them” with multiple choices for its value labels, including “strongly agree”, “agree”, “disagree”, and “strongly disagree” (see Table 2). Each choice in the value labels indicates an attitude toward the solution to the environmental problems. Therefore, this variable is classified as “attitude”. Similarly, the variables V41 and V49 in WVS3; V35, V36 and V37 in WVS4; V104 and V107 in WVS5; and V81 in WVS6 can also be put into the “attitude” family. The “action” family consists of V33, V38, V39, V42, V43, V44, V45 and V46 in WVS3; V33 and V34 in WVS4; V29, V105 and V106 in WVS5; and V30, V82 and V83 in WVS6. This family expresses the actions about whether the interviewees want to do something to reduce emissions or protect the environment. For instance, the variable V38 in WVS3 shows the wills of the interviewees to act on environmental protection according to the variable label “I would agree to an increase in taxes if the extra money were used to prevent environmental damage” and the corresponding value labels with four choices that are the same as those for the variable V40 (see Table 2). Therefore, V38 is put into the “action” family. Based on the implications of the variable labels and value labels, other variables including V33, V39, V42, V43, V44, V45 and V46 in WVS3; V33 and V34 in WVS4; V29, V105 and V106 in the WVS5; and V30, V82 and V83 in WVS6 are classified as “action” variables.

### Quantitative classification

In the second step, the hierarchical clustering analysis method is applied to quantitatively classify the variables based on the original WVS data. This step intends to confirm the results of the subjective classification from the first step.

Clustering analyses are conducted based on the variables of the WVS datasets. In each WVS dataset, a variable *X*_*j*_(*j* = 1, 2, ⋯, m) is defined as a vector:

1 $$ {X}_j=\left[\begin{array}{c}\begin{array}{c}{x}_{1j}\\ {}{x}_{2j}\\ {}\vdots \end{array}\\ {}{x}_{nj}\end{array}\right] $$

where the vector size *n* represents the number of valid respondents (see Table 1), *m* is the number of variables in the dataset (see Table 2 for the details: 11 in WVS3, 5 in WVS4, 5 in WVS5 and 4 in WVS6), and the element *x*_*ij*_ denotes the serial number of the choice from the *i*-th respondent among the value labels of the *j*-th variable (see Table 2). Because the answers are non-numerical values that cannot be used in arithmetical operations, the corresponding serial number is adopted as the numerical value of the element (Gelissen 2007; Hou et al. 2016); for example, 1 is used for “strongly agree,” 2 for “agree,” 3 for “disagree,” and 4 for “strongly disagree” (see Table 2). The serial number of the answer is continuous, which reasonably matches the choice from the value labels of the variable. All variables except V40 and V49 in WVS3, V35 and V37 in WVS4 and V30 in WVS6 obey a rule: the larger the number, the worse degree of recognition expressed by the answer (see Table 2). These five variables satisfy an inverse rule: the larger, the better.

In addition, the variables in the different WVS datasets have different sizes. For example, in WVS3, there are 40,222 valid respondents, while the WVS4 variables have a size of 27,534 (refer to Table 1). Therefore, it is very difficult to conduct a unified clustering over all selected WVS datasets. In this study, a strategy to separately cluster the WVS datasets one by one is used. The variables in each WVS dataset are quantitatively classified into the “action” and “attitude” families that were defined in the subjective classification. The clustering of the variables in each WVS dataset includes the following four steps:

1. Preprocessing

In the originally coded data in the WVS datasets, there are some meaningless choices in the value labels such as “Not asked,” “NA,” and “DK.” Additionally, some choices have negative serial numbers. Furthermore, as mentioned above, five variables have serial numbers of answers that do not satisfy the rule that all other variables obey. Therefore, it is necessary to preprocess the original data so that the meaningless choices and those with negative serial numbers are removed. Moreover, the inconsistencies in the serial number rule between the aforementioned five variables and the other variables are eliminated. Table 1 shows the numbers of valid countries and corresponding respondents (or cases) in different WVS datasets after preprocessing. The detailed information on the valid countries and cases are provided in Table 3.

**Table 3:** Names of valid countries and numbers of corresponding respondents in the selected WVS datasets

WVS3		WVS4		WVS5		WVS6	
Country	Case	Country	Case	Country	Case	Country	Case
Albania	383	Albania	690	Andorra	901	Algeria	867
Azerbaijan	1318	Argentina	886	Argentina	829	Azerbaijan	849
Argentina	709	Bangladesh	1275	Australia	1250	Argentina	908
Australia	1674	Bosnia	961	Brazil	1331	Australia	1323
Bangladesh	925	Canada	1636	Bulgaria	719	Bahrain	1068
Armenia	1320	Chile	1026	Canada	1539	Brazil	1386
Bulgaria	375	China	646	Chile	820	Belarus	1372
Belarus	1257	Indonesia	2	China	434	Chile	980
Chile	748	Iran	1004	Colombia	2293	China	2118
China	811	South Korea	344	Cyprus	605	Taiwan	990
Taiwan	536	Kyrgyzstan	232	Ethiopia	1293	Colombia	1351
Croatia	777	Mexico	721	Finland	912	Cyprus (G)	967
Czech Rep.	707	Moldova	821	France	923	Ecuador	1193
Dominican Rep.	273	Morocco	966	Georgia	1332	Estonia	1416
Estonia	703	Nigeria	1377	Germany	926	Georgia	1143
Finland	760	Pakistan	394	Ghana	1044	Palestine	894
Georgia	1576	Saudi Arabia	779	Guatemala	698	Ghana	1359
Germany	1483	Singapore	1344	Hong Kong	1033	Hong Kong	997
Hungary	478	Viet Nam	750	Hungary	888	India	4661
India	818	South Africa	804	India	899	Iraq	1052
Japan	322	Spain	856	Indonesia	1529	Japan	1689
South Korea	960	Sweden	721	Iran	1402	Kazakhstan	823
Lithuania	427	Turkey	2351	Iraq	2161	Jordan	1140
Mexico	1314	Uganda	831	Italy	639	South Korea	1167
New Zealand	583	Macedonia	815	South Korea	813	Kuwait	851
Nigeria	1502	Egypt	24	Malaysia	643	Kyrgyzstan	1416
Norway	1032	Tanzania	682	Mali	1424	Lebanon	1022
Peru	774	United States	1070	Mexico	1033	Libya	1708
Philippines	1066	Venezuela	239	Moldova	747	Malaysia	1228
Puerto Rico	915	Serbia	742	Morocco	1068	Mexico	1925
Romania	573	Montenegro	890	Netherlands	944	Morocco	849
Russia	1072			New Zealand	633	Netherlands	1717
Slovakia	722	Total	25,879	Norway	127	New Zealand	654
Slovenia	728			Peru	893	Nigeria	1708
South Africa	1895			Poland	938	Pakistan	1149
Spain	815			Romania	1476	Peru	1106
Sweden	740			Russia	1639	Philippines	1094
Macedonia	557			Rwanda	106	Poland	947
United States	1065			Viet Nam	717	Qatar	958
Uruguay	673			Slovenia	857	Romania	1446
Venezuela	766			South Africa	2395	Russia	2202
Serbia	765			Spain	957	Rwanda	1196
Montenegro	107			Sweden	808	Singapore	1530
Bosnia	538			Switzerland	1026	Slovenia	1048
				Thailand	1306	South Africa	3051
Total	37,542			Trinidad-Tobago	874	Zimbabwe	1422
				Turkey	1238	Spain	1127
				Ukraine	926	Sweden	1118
				Egypt	2762	Thailand	1112
				Great Britain	746	Trinidad-Tobago	938
				United States	170	Tunisia	987
				Burkina Faso	1391	Turkey	656
				Uruguay	778	Ukraine	1383
				Serbia-Monteneg	919	Egypt	1409
				Zambia	1149	United States	2120
						Uruguay	923
				Total	57,903	Uzbekistan	1306
						Yemen	936
						West Germany	906
						East Germany	1006
						Total	77,867

To clear up the inconsistencies in the rule as well as differ the rules for the two families, two different rules are used for the two families based on the subjective classification. All the “action” variables obey the rule: the larger the number is, the worse degree of recognition expressed by the answer, while all the “attitude” variables satisfy the inverse rule: the larger, the better.

2. Normalization

Table 2 shows that different variables may have different value labels, even in the same dataset, and the corresponding serial numbers have various maximum values. Therefore, it is necessary to normalize the elements of a variable using Z-scores (Richard and Audrey 1994) so that they are on the same scale:

2 $$ {x}_{ij}^{\prime }=\left\{\begin{array}{c}\frac{x_{ij}-{\overline{x}}_j}{S_j}\kern1.5em if\ {S}_j\ne 0\\ {}0\kern3.25em if\ {S}_j=0\end{array}\right.\kern0.75em \left(\genfrac{}{}{0pt}{}{i=1,2,\kern0.5em \cdots, n}{j=1,2,\cdots, m}\right) $$

where ${\overline{\mathrm{x}}}_j$ and *S*_*j*_ are the mean value and standard deviation of the *j*-th variable, respectively:

3 $$ {\overline{x}}_j=\frac{1}{n}{\sum}_{i=1}^n\;{x}_{ij},{S}_j=\sqrt{\frac{1}{n-1}{\sum}_{i=1}^n{\left({x}_{ij}-{\overline{x}}_j\right)}^2} $$

3. Definition of distance

The distance between any two variables *X*_*j*_ and *X*_*k*_ in the same WVS dataset is measured using their Pearson correlation:

4 $$ {r}_{x_j{x}_k}=\frac{\sum_{i=1}^n\left({x}_{ij}^{\prime }-{\overline{x}}_j^{\prime}\right)\left({x}_{ik}^{\prime }-{\overline{x}}_k^{\prime}\right)}{\sqrt{\sum_{i=1}^n{\left({x}_{ij}^{\prime }-{\overline{x}}_j^{\prime}\right)}^2{\sum}_{i=1}^n{\left({x}_{ik}^{\prime }-{\overline{x}}_k^{\prime}\right)}^2}} $$

The larger the correlation, the shorter the distance. According to (2) and (3), ${\overline{x}}_j^{\prime }=\frac{1}{n}{\sum}_{i=1}^n{x}_{ij}^{\prime }=0$, ${\overline{x}}_k^{\prime }=\frac{1}{n}{\sum}_{i=1}^n{x}_{ik}^{\prime }=0$, and $\sqrt{\sum_{i=1}^n{\left({x}_{ij}^{\prime }-{\overline{x}}_j^{\prime}\right)}^2{\sum}_{i=1}^n{\left({x}_{ik}^{\prime }-{\overline{x}}_k^{\prime}\right)}^2}=n-1$;thus, the formula to calculate the correlation coefficient can be simplified into

5 $$ {r}_{x_j{x}_k}=\frac{1}{n-1}{\sum}_{i=1}^n{x}_{ij}^{\prime }{x}_{ik}^{\prime } $$

4. Clustering and rescaled distance

**Fig. 1:**
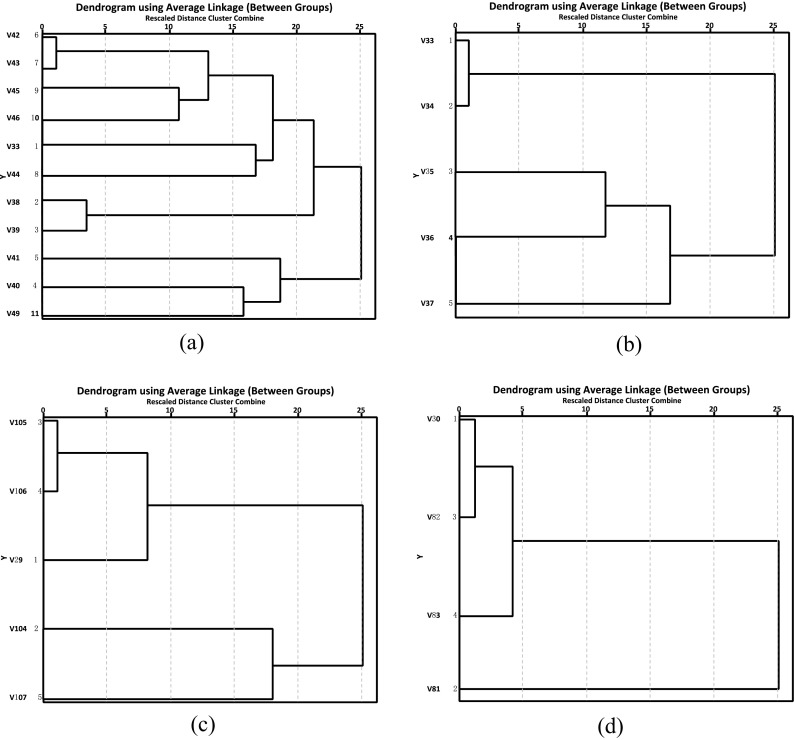
Hierarchical clustering of (**a**) WVS3, (**b**) WVS4, (**c**) WVS5, and (**d**) WVS6

The variables in the same WVS dataset are classified according to the distances among them. The distance between two clusters *C*_*p*_ and *C*_*q*_ is measured according to the average correlation coefficient between all inter-cluster pairs:

6 $$ {r}_{c_p{c}_q}=\frac{1}{n_1{n}_2}\sum \limits_{x_j\in {c}_p}\sum \limits_{x_k\in {c}_q}{r}_{x_j{x}_k} $$

where *n*_1_ and *n*_2_ are the numbers of variables in the clusters *C*_*p*_ and *C*_*q*_ , respectively. The “between-groups linkage” method, a kind of hierarchical clustering, is used for the classification, which works well for both elongated chain-type and clumpy type clusters (Zhang 2004). This method includes an iteration with *m*-1 steps. At the first step, each variable is regarded as a cluster, and there are a total of *m* clusters that can be combined into *m*(*m*-1)/2 inter-cluster pairs. The correlation coefficients between all inter-cluster pairs are calculated, and then the inter-cluster pair with the maximum correlation coefficient is combined to a new cluster. In this way, the number of clusters is reduced to *m*-1. A similar method is repeated until the number of clusters is reduced to 2 (see Fig. 1). If the maximum correlation coefficient at the first step is *r*_*M*_ and the minimum correlation coefficient at the last step of the iteration is *r*_*m*_, the (rescaled) distance is defined as ${D}_{c_p{c}_q}=l$ when ${r}_{l+1}<{r}_{c_p{c}_q}\le {r}_l$, where ${r}_l={r}_M-\left(l-1\right)\frac{r_M-{r}_m}{L-1}$ and *l* = 1, 2, ⋯, *L*, *L* + 1.

Figure 1 shows the hierarchical clustering results (here, *L* = 25). Among the 11 variables in WVS3, the variables V33, V38, V39, V42, V43, V44, V45, and V46 are sorted as “action” variables, and the variables V40, V41, and V49 are classed as “attitude” variables (see Fig. 1a). This classification is consistent with the subjective classification. For the classifications of the variables in the other WVS datasets including WVS4, WVS5, and WVS6, the results of the quantitative analyses are also the same as those from the subjective analyses (refer to Fig. 1b–d).

### Horizontal distribution of variables

As expressed in Eq. (1), each of the variables in the WVS datasets consists of tens of thousands of cases from many countries throughout the world. To obtain the horizontal distribution of a variable, the arithmetical means of the serial numbers of multiple choices from the respondents in the same countries are used so that each country has only one number. In this way, a variable *X*_*j*_ can be expressed as a new vector.

7 $$ {X}_j=\left[\begin{array}{c}{\tilde{x}}_{1j}\\ {}{\tilde{x}}_{2j}\\ {}\vdots \\ {}{\tilde{x}}_{pj}\end{array}\right],{\tilde{x}}_{lj}=\frac{1}{i_l-{i}_{l-1}}{\sum}_{k_{l-{1}^{+1}}}^{i_l}{x}_{kj}\left(l=1,2,\cdots, p\right) $$

where *p* is the number of countries, *i*_0_ = 0, and *i*_*l*_ − *i*_*l* − 1_ represents the number of questionnaire cases from the *l*-the country (please refer to Table 3). Because the original ranges of the serial numbers in different variables may be different, the new vector also requires normalization similar to Eqs. (2) and (3).

When the variables are expressed in a new form, it is unknown if the quantitative classification will be changed. To answer this question, similar hierarchical clustering analyses on the four datasets are conducted using the new vector form. The results show that the classification is the same as that from the “Quantitative classification” subsection, which reconfirms the classification from the previous subjective analysis and clustering analysis.

### Indices of action and attitude

After the efforts made in the “Subjective classification,” **“**Quantitative classification,” and “Horizontal distribution of variables” subsections, all variables in the selected WVS datasets were classified into two families, which are “action” and “attitude” with horizontal distributions. However, it is still difficult to quantify the families because each includes more than one variable in most cases, even in a specified WVS dataset that covers a specified period. Therefore, the main focus of this subsection is to quantify the above families and produce a continuous time series from 1994 to 2014.

The difficulty to produce a continuous and consistent time series of the two families is due to the inconsistency among the four periods (or 4 WVS datasets), i.e., the variables have quite different variable labels, sizes, and numbers in the different periods. Moreover, the variables are prone to multicollinearity and distorted estimations when highly correlated variables are directly analyzed by using statistical analysis. It is challenging to produce a continuous time series that is consistent among the four periods.

In this subsection, we attempt to use the EOF decomposition approach to determine the factors that are common to the two families from their own variables for each of the four periods. The first leading EOF mode of each family in each period is adopted as the common factor of the family in that period because this mode captures the most common feature of the variables in the family and is significantly correlated with most of these variables. As shown in Table 4, 12 of the 16 action variables have high correlation coefficients (greater than 0.66) with the first leading modes of action in different periods, while 8 of the 9 attitude variables are highly correlated (greater than 0.55) with the first leading modes in different periods. Almost all of the first leading modes have significant variance contributions (greater than 46%, see Table 5). The only exception is the first leading mode of the action family in the WVS3 dataset, whose variance contribution is only 35.4%. These leading modes basically show the primary meaning of all variables in the same family maximally.

**Table 4:** Correlation coefficients between the variables and the first leading modes of two families. (The emphasis indicates the high correlation coefficients with the first leading modes of action and attitude in different periods)

Dataset	Variable	First leading mode of the action family	First leading mode of the attitude family
WVS3	V33	*0.2961*	0.2007
	V38	*0.2807*	− 0.2470
	V39	*0.1736*	− 0.0928
	V40	− 0.1353	*0.6846*
	V41	− 0.3855	*0.5706*
	V42	*0.7941*	− 0.5015
	V43	*0.8625*	− 0.5493
	V44	*0.4561*	− 0.0354
	V45	*0.7797*	− 0.1488
	V46	*0.6654*	0.1791
	V49	− 0.1817	*0.7884*
WVS4	V33	*0.9465*	− 0.3797
	V34	*0.9465*	− 0.4477
	V35	− 0.2802	*0.7827*
	V36	− 0.5648	*0.7505*
	V37	0.0606	*0.4789*
WVS5	V29	*0.7440*	− 0.1965
	V104	− 0.1484	*0.7405*
	V105	*0.9285*	− 0.3652
	V106	*0.9564*	− 0.5076
	V107	− 0.4685	*0.7405*
WVS6	V30	*0.8930*	0.0102
	V81	− 0.0628	*1*
	V82	*0.8158*	− 0.0671
	V83	*0.8096*	− 0.1079

**Table 5:** Contributions of the first leading modes of the action and attitude families to the total variances in the selected WVS datasets

Dataset	First leading mode of the action family (%)	First leading mode of the attitude family (%)
WVS 3	35.4	47.2
WVS 4	89.6	46.8
WVS 5	77.7	54.8
WVS 6	70.6	100

It is understandable that the action family in the WVS3 dataset exhibits a low variance contribution because it is the largest family with 8 variables and may have much more leading modes than the other families, leading to the deconcentration of the variance contribution. Corresponding to this low variance contribution, 3 action variables (i.e., V33, V38, and V39) in WVS3 are correlated with the first leading mode of action with low coefficients (smaller than 0.3, see Table 4). This result indicates that this leading mode mainly reflects the characteristics of other action variables in WVS3; in particular, the correlation coefficients of the variables V42, V43, V45, and V46 are 0.7941, 0.8625, 0.7797, and 0.6654, respectively. Table 4 indicates that in each period (or each WVS dataset) the action (attitude) variables are always more highly correlated with the first leading mode of action (attitude) than with the first leading mode of attitude (action), which testifies the reasonability and correctness of the quantitative clustering. Finally, the indices of the two families during the periods 1999–2004, 2005–2009, and 2010–2014 are defined as the first leading modes of the corresponding families.

## Analysis of “action” and “attitude” indices

**Fig. 2:**
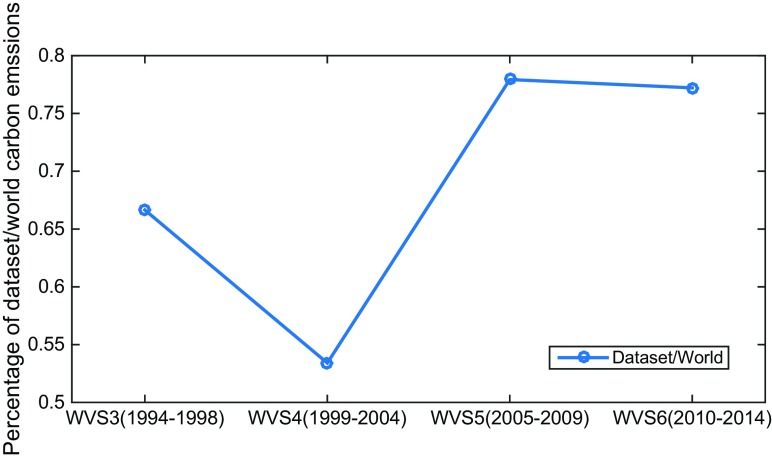
The contribution rates of the countries included in the WVS datasets to global carbon emissions

Prior to analysis, the representativeness of the countries included in the 4 WVS datasets should be investigated to ensure reasonability. For this purpose, the total contribution rate of the countries in each WVS dataset to the global carbon emissions in the same period is calculated, and the results are shown in Fig. 2. The carbon emissions by the countries account for 66.65% of the global carbon emissions in WVS3, 53.41% in WVS4, 77.93% in WVS5, and 77.21% in WVS6. Thus, the countries included in these 4 WVS datasets are all representative.

**Fig. 3:**
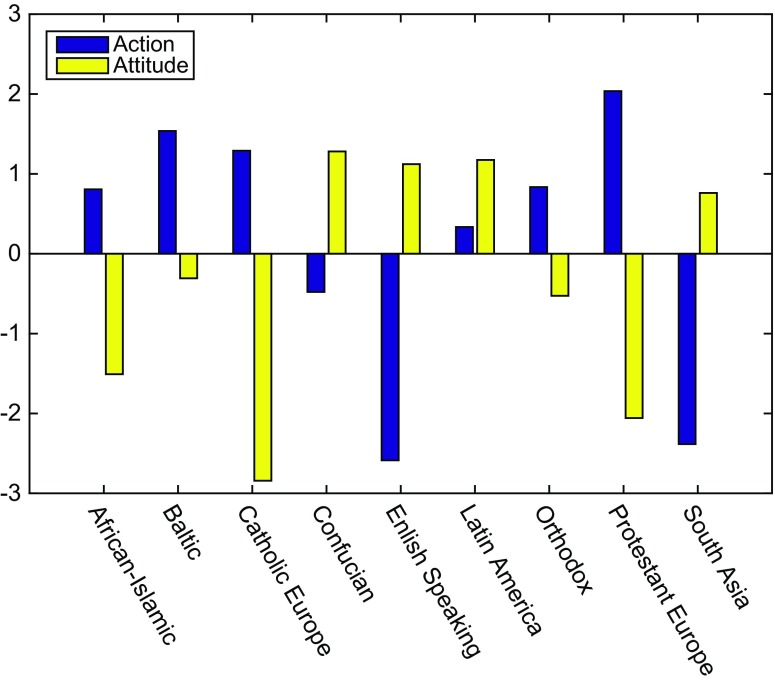
Actions and attitudes toward environmental protection in different cultural districts

Although very few countries have values data in all four periods (see Table 3), each cultural district has complete data in these periods (Fig. 3). The spatial distributions of the action and the attitude variables can be obtained in terms of the cultural district (Fig. 3) by computing the average value over each cultural district in the world WVS cultural maps and over the four periods. The cultural maps are different in different periods due to the change of the country position, but overall, the religion of the country does not change. Fig. 3 indicates that English-speaking countries and South Asia exhibit the best environmental protection action, followed by Confucian countries. In addition, European and Baltic countries exhibit the worst action and attitude. According to Fig. 3, the top three areas in terms of attitudes to environmental protection are the Confucian, Latin American, and English-speaking countries, followed by South Asian countries. In addition, the Catholic nations of Europe are the worst, followed by the Protestant countries of Europe and Africa-Islamic countries. Figure 3 also shows that the English-speaking countries and South Asia show better actions and attitudes, while the Protestant and Catholic European countries are worse on both. Latin America presents bad action but good attitude.

For English-speaking countries, it is very common to think that the individual ownership, self-expression and civil rights are important. These countries have an open culture and prefer to accept new things, and the wealth of these countries makes them willing to use the most advanced technology to improve the quality of life, and a good environment is one of the key parts of a high-quality life. Therefore, these countries have a good environmental protection action (Inglehart 1977, 1990, 1995, 1997). In addition, at the same time, lifestyles that depend on machines and automobiles also increase energy consumption. The environmental pressure objective contributes to the upgrade of their environmental attitudes.

Generally, people are likely to be engaged in pro-environmental behavior when the environmental problems have threatened their health, social stability, animals, and plants. This viewpoint comes from the value-belief-norm (VBN) theory (Stern 2000; Stern et al. 1995, 1999). The effect of religion in South Asia (e.g., India) indicates that their wishes are to be consistent with nature; thus, the power consumption is far lower than the international standard (Bhattacharyya, 2007). Religiosity is positively correlated with traditional values and negatively correlated with hedonism values (Schwartz and Huismans 1995). Adverse environmental conditions largely promote the South Asia districts to show better actions and attitudes.

The Confucian region exhibits the best environmental attitude. The core of Confucian culture is “benevolence,” which includes thinking human beings are part of nature, and nature is the mother that should be cared for (Chen 2003). This culture easily leads to a positive environmental protection attitude in this region. Moreover, most countries in this region are developing countries and their economic developments have resulted in environmental destruction to some extent. The objective environment pressure and the enhanced environmental law enforcement due to the promotion of public participation in these countries further improve their attitude. Because the Confucian cultures emphasize self-cultivation and self-control (Fei 2013; Sungmoon 2009) along with ambient pressure, the Confucian countries present good actions. At the same time, the Confucian culture is of sociality, and there are the mentalities of “free ride” and “the bird out of the group will be shot”. Therefore, the behavior measures are still lower than the awareness of environmental protection in this region.

The WVS data for the European countries show an unexpected result in terms of the bad attitude and action on environmental protection in these countries. As noted by Bernd (2015), European culture is influenced by the early Renaissance, which is human-centered and advocates individual liberation, equality, and freedom. In particular, material enjoyment and luxury were pursued later in the Renaissance period. Most sociologists believe that the fate of the religion in Europe will not be able to curb the decline after the 19th and 20th centuries, which indicates the growing trend of secularization (see European Studies on Religion and State Interaction: http://www.euresisnet.eu/Pages/ReligionAndState/GERMANY.aspx). Therefore, the European action and attitude of environmental protection will be less affected by their religions. On the other hand, the lower environmental pressure in Europe due to the highly developed economy might be another reason for this result.

The environmental attitudes and behaviors are not good in any of the Africa-Islamic countries. These countries are relatively lacking in wealth and occasionally face unstable factors (McCormack 2005). According to Franzen (2003), different affluence levels result in different levels of environmental concern from the people. Those countries that are not affluent cannot reallocate scarce economic resources to achieve environmental goals, and their primary concerns are the survival of their own families.

## Conclusions

A total of 25 variables chosen from the four WVS datasets were classified into two families by using subjective and clustering analyses to reflect behavior and motivation, respectively. Their spatial distributions were obtained in each cultural district according to the World Culture Map. These distributions were preliminarily analyzed and explained by considering the cultural, law, and environmental conditions as well as economic development in different countries and regions.

The results show that different cultural districts have different actions and attitudes toward environmental protection. These differences are due not only to objective factors such as environmental pressure, resource endowments, and economic development but also to subjective factors, e.g., cultural and religious factors. English-speaking countries have an open culture with innovative technology, making them willing to use the most advanced technology to change human life. Therefore, these countries exhibit good environmental protection actions. At the same time, high-level energy consumption results in intensive carbon dioxide emissions. The historical environmental pressures and well-established education systems contribute to the elevation of environmental attitudes. The religions in South Asia have restricted the behavior of people, and they show environmentally friendly actions and attitudes. The Asian Confucian cultures emphasize self-cultivation and self-control, leading to good attitudes and actions. The results of the countries in Continental Europe present unexpected reverse relations between attitudes and actions in terms of environmental protection, which is very likely due to the lesser impact of environmental pressure. In African-Islamic countries, great effort is still required to enhance the attitudes regarding environmental protection because overcoming poverty and social instability is still the primary concern for family survival.

Attitudes and actions are highly associated with the patterns and stages of regional economic development. For example, many developed countries in different cultural regions (e.g. the UK, the USA, and Japan) followed the same path from agriculture, manufacturing to services and experienced environmental degradation and remediation. This phenomenon means that in many cases, economic laws can have larger roles than moral and cultural impacts on driving human actions regarding environmental protection. Policymaking in many developing countries should pay attention to the economic effects on both attitudes and actions. In particular, improving education systems may be the best way to stress the positive aspects in regional cultural traditions, increase public awareness of environmental protection, learn lessons from other countries or cultural systems, and achieve environmental technology transfer. In this direction, a future line of research might investigate the key factors, including economic growth and other variables that affect environmental protection values.
